# Supplementary figures and images for: The Stochastic Early Reaction, Inhibition, and late Action (SERIA) model for antisaccades
Source: PLoS Comput Biol. 2017 Aug 2;13(8):e1005692. doi: 10.1371/journal.pcbi.1005692 (PMC5555715; doi:10.1371/journal.pcbi.1005692)

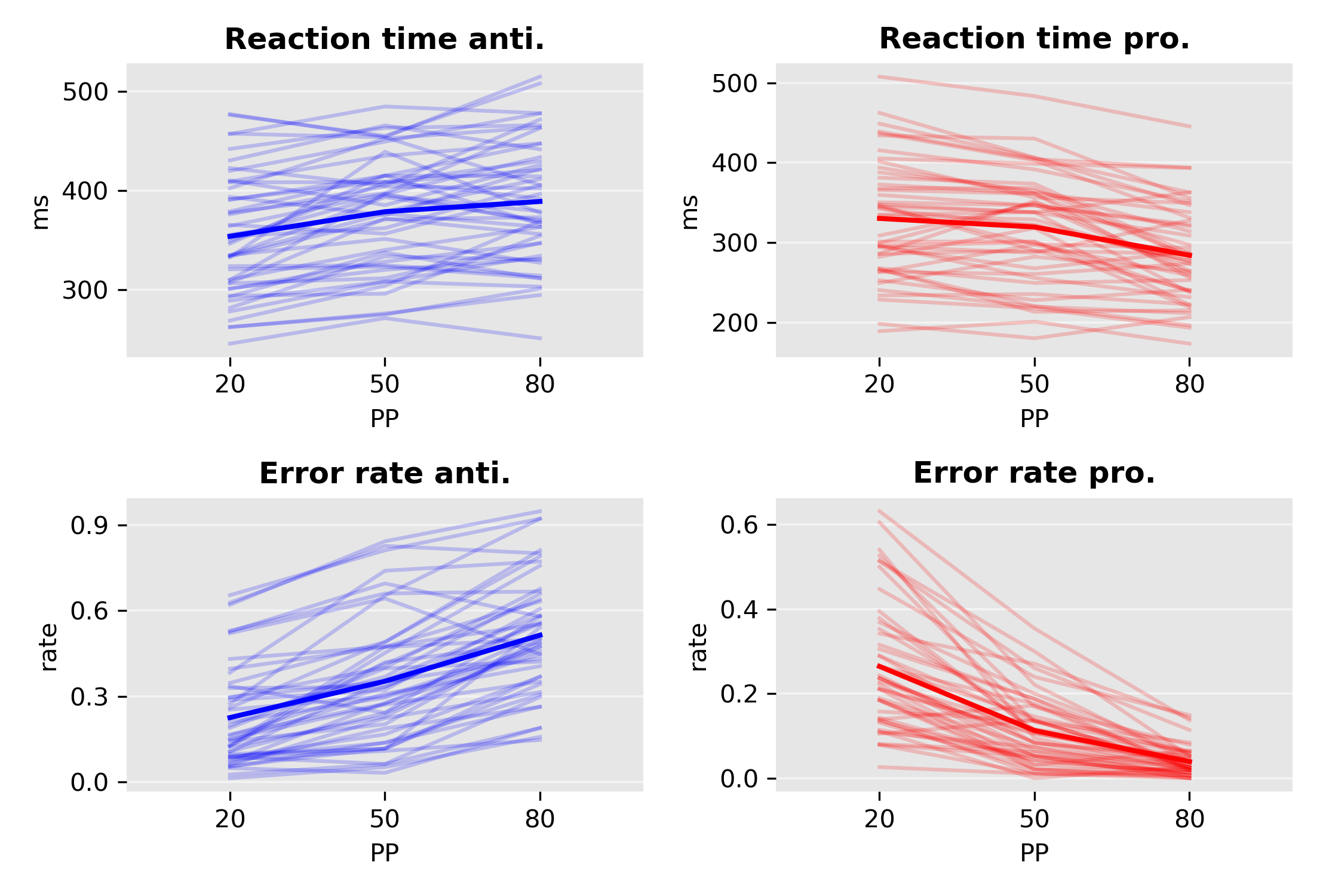

Supplement: S1 Fig — Mean reaction times and error rates are displayed as solid lines. (TIFF) [file pcbi.1005692.s002.tiff]
